# Prediction of Chromatin Accessibility in Gene-Regulatory Regions from Transcriptomics Data

**DOI:** 10.1038/s41598-017-04929-6

**Published:** 2017-07-05

**Authors:** Sascha Jung, Vladimir Espinosa Angarica, Miguel A. Andrade-Navarro, Noel J. Buckley, Antonio del Sol

**Affiliations:** 1 Luxembourg Centre for Systems Biomedicine (LCSB), University of Luxembourg, Belvaux, Luxembourg; 2 Faculty of Biology, Johannes-Gutenberg University of Mainz, Mainz, Germany; 3 Institute of Molecular Biology, Mainz, Germany; 4 Department of Psychiatry, Warneford Hospital, University of Oxford, Oxford, United Kingdom

## Abstract

The epigenetics landscape of cells plays a key role in the establishment of cell-type specific gene expression programs characteristic of different cellular phenotypes. Different experimental procedures have been developed to obtain insights into the accessible chromatin landscape including DNase-seq, FAIRE-seq and ATAC-seq. However, current downstream computational tools fail to reliably determine regulatory region accessibility from the analysis of these experimental data. In particular, currently available peak calling algorithms are very sensitive to their parameter settings and show highly heterogeneous results, which hampers a trustworthy identification of accessible chromatin regions. Here, we present a novel method that predicts accessible and, more importantly, inaccessible gene-regulatory chromatin regions solely relying on transcriptomics data, which complements and improves the results of currently available computational methods for chromatin accessibility assays. We trained a hierarchical classification tree model on publicly available transcriptomics and DNase-seq data and assessed the predictive power of the model in six gold standard datasets. Our method increases precision and recall compared to traditional peak calling algorithms, while its usage is not limited to the prediction of accessible and inaccessible gene-regulatory chromatin regions, but constitutes a helpful tool for optimizing the parameter settings of peak calling methods in a cell type specific manner.

## Introduction

The differential gene expression patterns of cells are established by different regulatory landscapes at the transcriptional and epigenetic layers. The dynamic epigenetic landscapes of cells shape different regulatory scenarios by changing the accessibility and activity of chromatin regions, determining different transcription factor (TF) binding landscapes and gene regulatory networks^1, 2^. Moreover, the chromatin landscape is established and maintained by the binding of transcriptional regulators to specific genomic regions^3–5^. Chromatin structure dynamics is essential for the regulation of niche-cell interaction^6^ and many phenotypic transitions, such as cellular differentiation and reprogramming^7–9^ or disease onset and progression^10, 11^. Recently, great efforts have been devoted to the experimental profiling of the epigenetic states in different cell types^11–13^ and chromatin dynamics during complex biological processes^6, 9^.

Different studies have shown that active regulatory elements are located in accessible, i.e. nucleosome depleted, chromosomic regions^14–18^ and chromatin accessibility is predictive of functional activity within a specific cell type^16^. To date there exist several experimental methods for profiling nucleosome depleted chromatin regions. In particular, DNase hypersensitivity, formaldehyde-based FAIRE, or assay for transposase-accessible chromatin using sequencing (ATAC-seq)^15, 19, 20^ are frequently used to pinpoint genomic regions containing regulatory binding sites that are functional in each specific cell type or condition^6, 9, 18^. However, computational methods used for identifying genomic regions enriched with aligned reads – i.e. peak callers – have important limitations and, depending on the method used, the chromatin accessibility assignments can be significantly different after processing the same dataset. In a previous study comparing the called peaks obtained using four of the most widely used algorithms (Hotspot^16, 21^, F-Seq^22^, Zero-Inflated Negative Binomial Algorithm (ZINBA)^23^ and Model-based Analysis of ChIP-Seq (MACS)^24^) it was found that the overlap of the results obtained by different methods was rather low, corresponding to only 11% of the total called peaks^25^. Moreover, this study also proved that the selection of the parameters used by each peak-caller has significant effects on the genome wide accessibility profile obtained in each case^25^ whereby an optimal setting of the parameters is usually not known a priori. Namely, the parameterization used for controlling the false discovery rate of the peak callers is key, as more stringent cutoffs render increased false negative rates, while less stringent cutoffs result in increased false positive rates. Furthermore, repetitive and low-mappable regions further increase the number of false negative peaks and can only be assessed empirically^13^. Hence, there is a need for computational approaches for predicting chromatin accessibility that are less parameter sensitive in order to overcome the limitations of current peak-callers and provide a rationale for linking the expression of the genes related to a specific phenotype with the corresponding chromatin accessibility landscape.

In this paper we present a methodology for performing predictions of chromatin accessibility at gene-regulatory regions from transcriptomics data. We trained a hierarchical random forest model from ENCODE gene expression and chromatin accessibility data, encompassing an ample dataset of different human cell types. After deriving the classification model from RNA-seq expression data, we performed a thorough validation of our method to predict chromatin accessibility based on a gold standard dataset compiled from TF and histone modification ChIP-seq experiments. This analysis accentuates the clear improvements of our predictions compared to peaks obtained from the most commonly used peak callers (MACS, Hotspot and F-Seq) regardless of the applied false discovery rate thresholds. Furthermore, we show that the recall of our predictions and called peaks in gene-regulatory regions is able to identify the most accurate peak calling parameters with respect to the gold standard dataset.

Thus, these results indicate that our method for predicting accessible and inaccessible gene-regulatory chromatin regions is able to assist currently available peak calling algorithms in overcoming and addressing their limitations. In particular, our method not only can predict the accessibility of gene-regulatory regions, but it can also optimize the parameters of current peak calling algorithms.

## Results

### Hierarchical classification model construction and cross-validation

Here, we present a methodology for predicting chromatin accessibility solely based on transcriptomics data, which is not dependent on the complex parameterizations needed by most current peak calling algorithms. Namely, our method predicts accessibility of genes based on a hierarchical classification tree model trained with datasets including experimentally derived gene expression and chromatin accessibility data. We aligned chromatin accessibility data, i.e. DNase-seq data, to human genome version 19 (hg19) and subsequently derived regions of local enrichment (hotspots) using the Hotspot algorithm^21^ with 1% false discovery rate. A previous study identified that expressed genes show different distributions of their overlapping DNase-seq peaks. While highly expressed genes contain peaks around their transcription start and end sites, medium and lowly expressed genes predominantly contain peaks in the gene body^26^. Following this rationale, we obtained a binary accessibility assignment for each gene by calling it open if at least one hotspot is overlapping its promoter or gene-coding region and closed otherwise. In order to train the model we prepared a dataset comprising RNA-seq and DNase-seq data of 18 distinct human cell types or cell lines. The predictions of the fitted model are hierarchically combined using classification tree boosting methods (see Methods).

After training the model, we assessed how its predictions generalize to an independent dataset by performing Leave-one-out cross-validation. Therefore, the dataset of 18 human cell types and cell lines was partitioned by either chromosomes or cell types and cell lines of which all but one partition constitute the training data and the other one the validation dataset. The results of the Leave-one-out cross-validation were subsequently compared to the predictions made by the full model to evaluate the generalizability of the model. In all of the Leave-one-out experiments that we conducted, we observed a Pearson correlation coefficient greater than 0.95 with respect to the predictions of the full model, which supports the proposed generalizability to independent datasets (see Supplementary File S1 for a detailed presentation of the results). In addition to these analyses, we analyzed the reproducibility of the results with respect to different expression replicates of the same cell type or cell line. We obtained a second replicate for 17 cell type/line included in the training dataset produced from the same lab, trained the model with one replicate, predicted the accessibility for both replicates and assessed their Pearson correlation. Median correlations of 0.91 and 0.88 when training with the first and second replicate, respectively, underline a high reproducibility of the results in the presence of multiple replicates. Of note, the correlation of the different gene expression replicates is on average even higher (median: 0.97) but contain three outliers (0.92, 0.91 and 0.7). These outliers are likely to influence the overall correlation of our predictions and seem to be causal of the lower, but still very high, correlations of the predictions (see Supplementary File S1 for a detailed assessment).

Importantly, the training dataset of 18 human cell types and cell lines contains several cancer cell lines, which typically harbour many structural variations^27, 28^. Therefore, reads from RNA-seq and DNase-seq experiments from cancer cell lines should not be aligned to the reference genome but rather to the sequenced genome of the cancer cell line under consideration. However, these sequenced cancer genomes are typically not available, which only allows the alignment to the reference genome. Due to this limitation, we also aligned all reads from cancer cell lines to the hg19 reference genome and trained the model with this data. In order to quantify the influence of the cancer cell lines on the predictions, we trained the model with the full dataset and non-cancer cell lines only and assessed the correlations of the predictions for the non-cancer cell lines. The predictions of the two models showed consistently high correlations of approximately 0.93 (standard deviation: 0.006) and as such underline the negligible influence on the predictions. However, in general, the effect of structural variations in cancer cell lines on the predictive accuracy of the model can be hardly quantified and thus the incorporation of cancer cell lines has to be taken with care.

### Comparison of the chromatin accessibility prediction strategy against traditional peak calling methods

In order to assess the predictive accuracy of our methodology, we compiled gold standard datasets for six cell lines (A549, GM12878, H1-hESC, HeLa-S3, HepG2 and K562) from ENCODE^13^ having the most experimental data and compared our predictions to called peaks obtained with F-Seq^22^, MACS^24^ and Hotspot^21^. Each gold standard dataset is composed of cell type specific sets of accessible and inaccessible genes. First, genes in accessible regions are obtained from significantly enriched regions of cell type specific transcription factor binding site (TFBS) ChIP-seq, since previous studies identified TF binding sites to be predominantly located in DNase hypersensitive regions^15^. We compiled 31 to 100 TFBS ChIP-seq experiments from ENCODE^13^ and identified genes overlapping with at least one TFBS, which would be a strong indication that it is encoded in a chromatin accessible region. Inaccessible genes, however, cannot be reliably determined by TFBS ChIP-seq experiments since the absence of binding events could be an indication that the gene is coded in a heterochromatic region in the corresponding cell line, or a lack of information of ChIP-seq binding site information for other TFs that could bind in this region. Thus, in order to overcome this problem we determined the gold standard dataset of inaccessible genes using ChromHMM^29^, a computational tool widely used to integrate epigenetics experimental data to segment the genome into chromatin states. We use precompiled datasets from the Roadmap Epigenomics project^11^ including five histone modifications – i.e. H3K4me1, H3K4me3, H3K36me3, H3K9me3 and H3K27me3 – and, according to this data, regions showing enrichment in (i) a combination of H3K4me3, H3K36me3^30^ and H3K9me3 – indicative of ZNF genes and repeats that are targets of heterochromatin proteins^31, 32^ – or (ii) H3K9me3 alone are considered to be heterochromatic. Similarly to the processing of TFBS ChIP-Seq experiments, genes overlapping heterochromatic regions will correspond to the set of inaccessible genes in the gold standard set.

**Figure 1:**
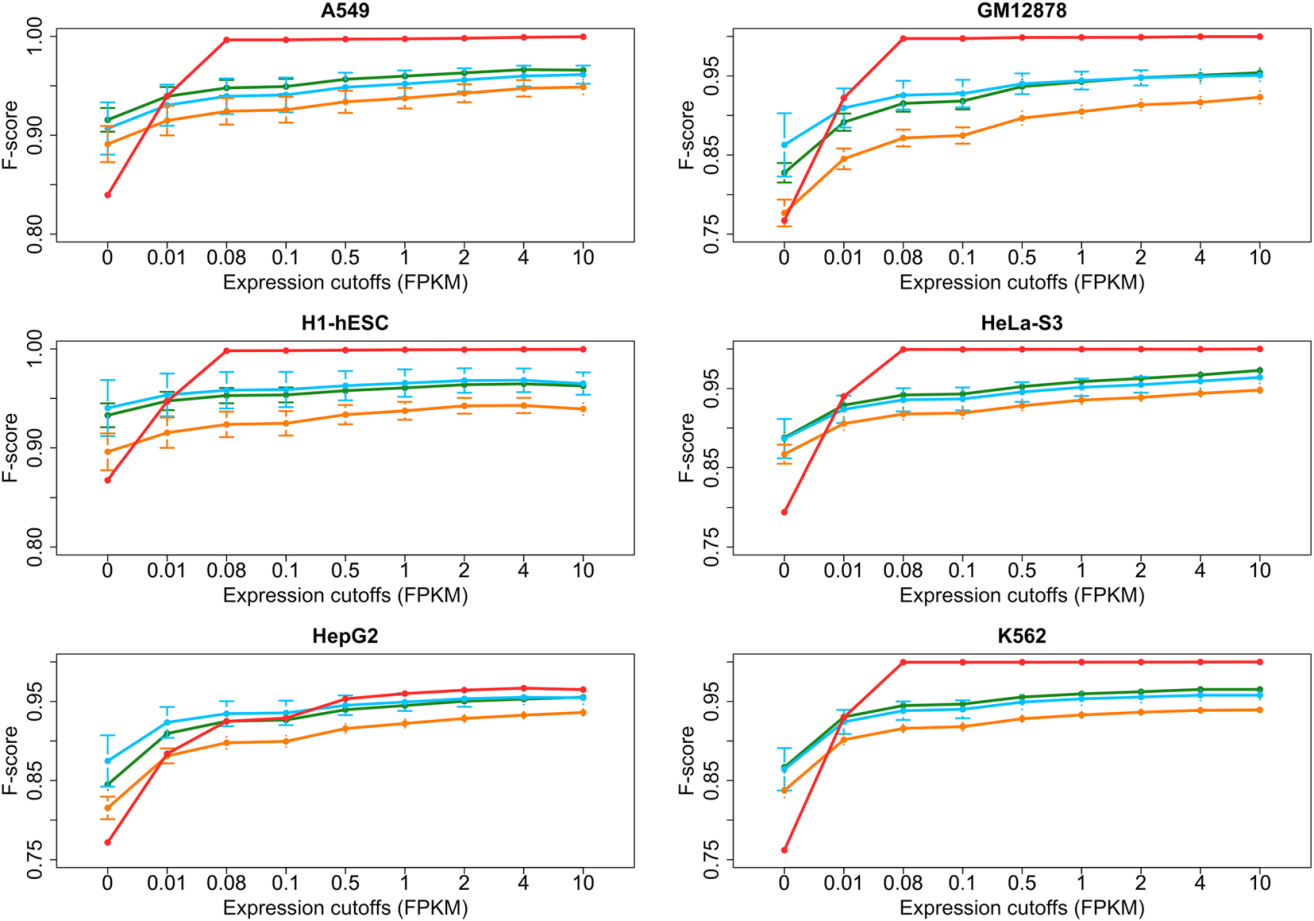
${F}_{1}$-Scores after applying FPKM expression cutoffs in each gold standard dataset. Comparison of ${F}_{1}$-scores after applying lower expression cutoffs in all six gold standard datasets of our predictions (red line), F-Seq (blue line), MACS (orange line) and Hotspot (green line). Dots, exemplifying mean values, and confidence intervals are shown, representing ${F}_{1}$-scores across replicates and FDR (z-score) cutoffs. Only genes that are more expressed than the cutoff were taken into account for the calculation of ${F}_{1}$-scores (0 cutoff representing all genes). While our method shows on average 0.075 lower scores for the whole dataset, the performance increases when only non-expressed genes are omitted and gradually increases to 0.99 in all cases except HepG2.

For comparing peak calling algorithms against our predictions, we obtained DNase-seq experimental data for each gold standard cell line (one replicate for H1-hESCs and two replicates for A549, GM12878, HepG2, HeLa-S3 and K562) and identified DNase hypersensitive sites (peaks) for various false discovery rate or Z-score thresholds for MACS, F-Seq and Hotspot to determine accessible and inaccessible genes (see Methods for details). We applied our methodology to publicly available RNA-seq for the six gold standard cell lines and obtained accessibility predictions. Predictions and accessibility assignments from peak calling methods – in the following referred to as observations – were then compared against the gold standard datasets by means of the harmonic mean of precision and recall, the ${F}_{1}$ – score. Since our method utilizes, in contrast to traditional peak calling algorithms, transcriptomics data to classify genes in accessible and inaccessible chromatin regions, we assessed how misclassified genes are distributed over the range of gene expression values to obtain information about the performance. Therefore, we applied lower FPKM expression thresholds, i.e. only taking into account genes that are expressed above the selected threshold, and examined changes of the ${F}_{1}$- score (Fig. 1).

When analyzing the complete datasets, which corresponds to a threshold of 0 FPKM, the predictions provided by our methodology obtain on average 0.065 (A549), 0.055 (GM12878, H1), 0.086 (HeLa-S3), 0.073 (HepG2) and 0.094 (K562) lower scores in comparison to peak calling algorithms. A possible explanation is provided by the deterministic behavior of the hierarchical classification tree where all genes with the same expression value are assigned the same accessibility status. We thus observe many false negative assignments in our predictions for genes that are not expressed at all (0 FPKM). However, ${F}_{1}$- scores above 0.75 indicate a good performance of our predictions. After applying different thresholding criteria, i.e. excluding genes with expression values below the threshold, the performance of our method gradually increases and obtains ${F}_{1}$ – scores of 0.999 for genes that are more expressed than 0.08 FPKM in all datasets except HepG2. In particular, genes that show expression values of more than 0.08 FPKM, which is considerably lower than typical thresholds for defining expressed genes^33–35^, are accurately predicted by our methodology. It is to note that 0.08 FPKM does not serve as a fixed threshold above which all genes are considered to be accessible but only reflects the lowest expression value above which the computed ${F}_{1}$-scores are close to the optimum. Due to the hierarchical classification tree model, genes above this value can still be classified as inaccessible.

Of note, the cell lines included in the gold standard datasets are also part of the training dataset, which might influence the results of our model and mask potential overfitting issues. We therefore did not use DNase-seq data in the gold standard datasets but distinct TFBS and Histone modification ChIP-seq experiments for assessing the performance of our approach to ensure the independence of the training and validation datasets. Furthermore, the results described above clearly reject overfitting of our method. The ${F}_{1}$ – scores do not correlate with those of Hotspot, the peak calling method with which the training samples were processed, and are also significantly better than for F-Seq and MACS. Further support is provided by the Leave-one-out cross-validation of our model that were presented in the last section. This assessment shows that the predictions of our model are highly consistent when leaving out one of 18 training samples. Thus, the ${F}_{1}$- scores would be highly similar as well when performing the predictions without the cell line in the gold standard dataset, which supports that the results on the considered gold standard datasets are not biased by the fact that the expression of these cell lines are also included in the training set.

Since the predictions obtained with our model and the observations differ for a fraction of genes, we investigated how many of the genes predicted to be open but not declared open in the observations can be validated by the transcription factor binding events. We considered the TFBS ChIP-seq experiments for the six cell lines in the gold standard dataset and found that between 0.47 and 0.67 (median: 0.62) of those genes are bound by a transcription factor and, thus, should be considered accessible (see Supplementary Fig. S1). Considering that the gold standard dataset consists of only 31 to 100 TFBS ChIP-seq experiments per cell lines, these numbers are likely to increase when more ChIP-seq experiments become available for these particular cell lines and support the increased performance of our method for predicting accessible gene-coding chromatin regions.

Overall, the results of our analysis underline that gene expression is an accurate predictor of chromatin conformation for both accessible and inaccessible regions.

**Figure 2:**
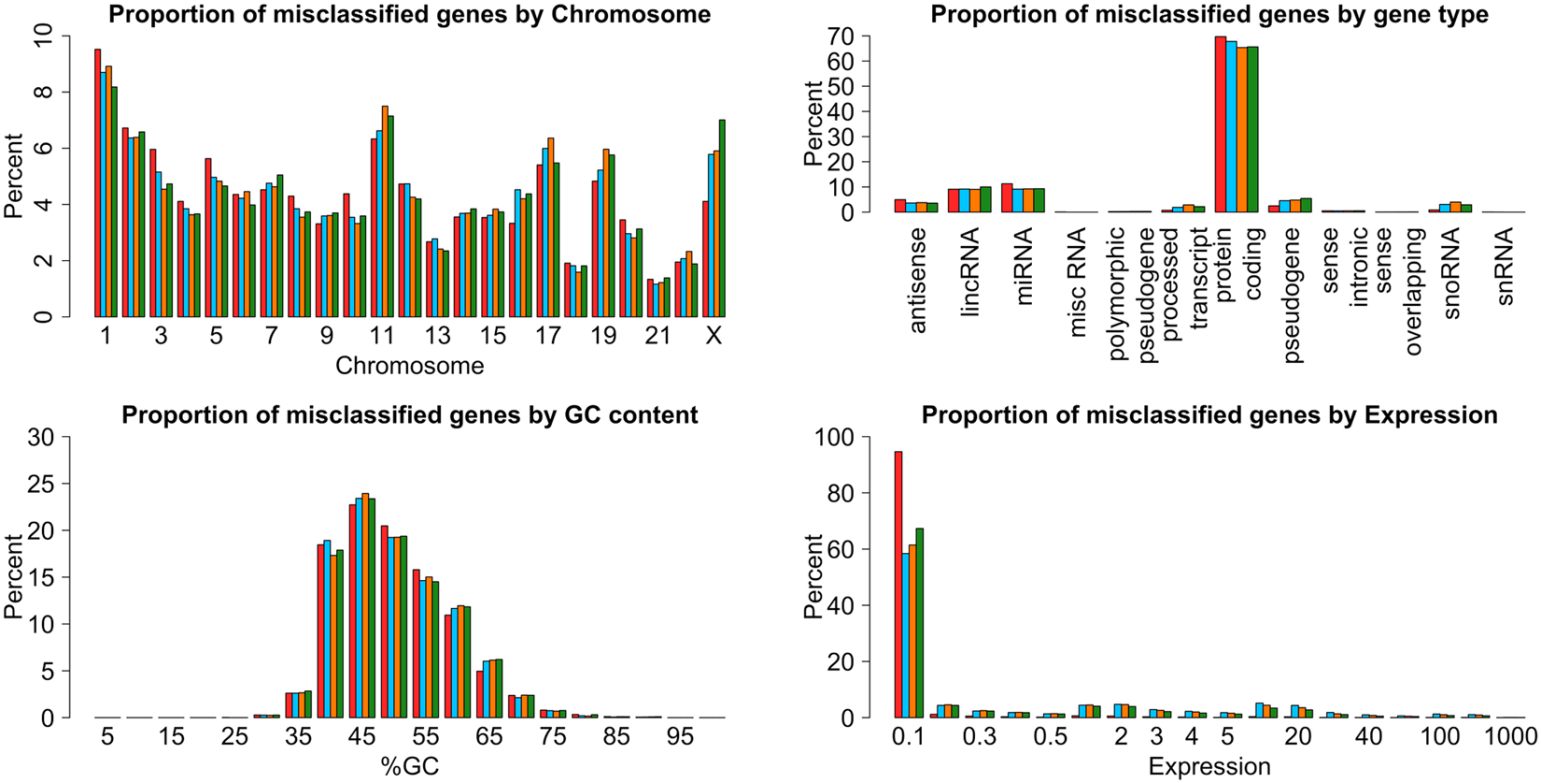
Comparison of our method to peak callers with respect to potential sources of bias. Potentially influencing factors for our predictions (red bars) where compared against F-Seq (blue bars), MACS (orange bars) and Hotspot (green bars). Neither, the chromosomal location nor gene type nor the GC content of misclassified genes distinguishes predictions from observations. However, by predicting accessibility based on gene expression (bottom right histogram), we significantly reduce the number of misclassified genes in regions expressed above 0.1 FPKM (6% compared to 41.6%, 38.6% and 37.2%). Due to the deterministic prediction, genes with no expression are overrepresented in the range of 0 to 0.1 FPKM (first bar, 38% out of 94%).

Utilizing gene expression as a predictor of chromatin accessibility, however, potentially introduces biases in the predictions in comparison with peak calling algorithms. First, the GC content of genes is negatively correlated with their methylation level^36^, thus, high methylation levels are indicative of low/no expression although the gene is located in accessible chromatin and is more likely to be misclassified. However, inspection of the GC content of misclassified genes by F-Seq (blue bars), MACS (orange bars), Hotspot (green bars) and our predictions (red bars) shows no significant differences (Fig. 2). Second, the abundance of RNA transcripts is divergent for different gene types. Previous studies showed that protein-coding genes are, for example, on average more expressed than non-coding genes^37^. In particular, the maximum expression of the majority of non-coding genes is below 1 FPKM and as such these genes are hypothetically more likely predicted to be located in heterochromatic regions^38^. Our data rejects this hypothesis as the percentages of misclassified genes per gene type is less than 7% for protein-coding genes and less than 3% for all other types (Fig. 2). Third, we examined a potential bias of our method with respect to peak calling algorithm in the chromosomal location of genes. This analysis serves as a negative control in which no bias is expected. Indeed, our assessment confirms that by showing a maximum difference of 3% per chromosome (Fig. 2). At last, we examined the distribution of misclassified genes by F-Seq, MACS, Hotspot and our predictions throughout the complete expression range and observed a significantly skewed distribution towards lowly expressed genomic regions when using our methodology. While the amount of misclassified genes is significantly higher for predictions in genes expressed below 0.1 FPKM – i.e. 94% of all misclassified genes are expressed between 0 and 0.1 FPKM, compared to 58.4%, 61.4%, 67.3% by F-Seq, MACS and Hotspot, respectively – the number of misclassified genes predicted with our methodology for highly expressed genes is rather low (Fig. 2). In the context of binary gene expression, typical thresholds between 0.1 and 1 FPKM are used to classify whether a gene is expressed or not^33–35^. In these regions, however, utilizing our method constitutes a clear improvement having only up to 3% misclassified genes in each segment compared to, on average, 14.4% of peak calling algorithms (Fig. 2).

Overall, our methodology is not only able to more accurately classify genes in accessible and inaccessible chromatin conformation by using gene expression data but also shows no bias towards gene types, GC content or chromosomal location. The most significant difference of our method compared to traditional peak calling algorithms, however, is reflected in the percentage of misclassified genes across the gene expression range. In this case, our method misclassifies only 6% of genes with expression values higher than 0.1 FPKM – a very low threshold to define expressed genes – compared to 14.4% of traditional peak calling methods.

### Obtaining optimal peak calling parameters by comparing called peaks and predictions

The results described above demonstrated that our methodology can accurately identify genes located in accessible and inaccessible chromosomal regions. However, the hierarchical classification model – as it was trained – can only provide insights about the chromatin conformation of the whole gene but does not allow for further functional analysis of peak locations provided by peak calling methods. As already discussed in a previous study comparing peak calling methods for DNase-seq data^25^, the choice of parameters – and especially the false discovery rate threshold – plays an important role for obtaining reliable results.

**Figure 3:**
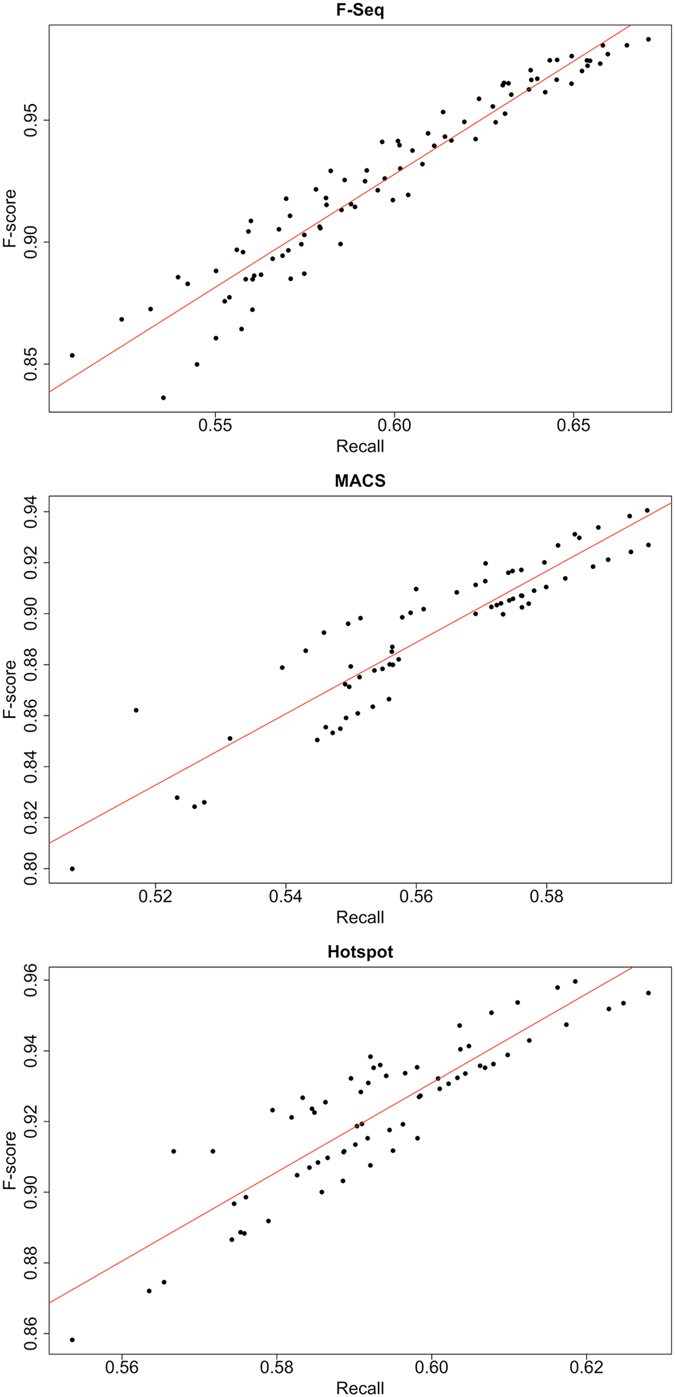
Linear Regression of ${{\boldsymbol{F}}}_{{\boldsymbol{1}}}$-scores and recall based on observations versus predictions. Linear regression (red lines) of ${F}_{1}$-scores and recall – computed between observations and predictions – for each peak caller proves their linear relationship (adjusted R squared of 0.92, 0.84 and 0.79, respectively). Each dot represents a tuple of FDR (or z-score in case of F-Seq) cutoff, cell line and replicate.

We thus sought to investigate how the predictions obtained by our methodology can help to identify the best parameter sets for F-Seq, MACS and Hotspot. In particular, we are interested in identifying parameter settings that maximize the ${F}_{1}$- score with respect to our gold standard datasets. The results described in the previous section showed that our method especially predicts accessible regions more reliably, which leads to the assumption that optimal peak calling parameters can be obtained by maximizing the agreement between called peaks and predicted accessible regions. This notion can be expressed as the recall of called peaks against our predictions for genes that are expressed above 0.01 FPKM. We varied the false discovery rate (Z-score) parameter for each peak caller (see Table 1) and evaluated the recall against our predictions as well as the ${F}_{1}$-score with respect to the gold standard datasets for each setting. Notably, we observe a strongly increasing, linear relationship between the obtained ${F}_{1}$-scores and the recall of called peaks and our predictions with adjusted R-squared values of 0.92, 0.84 and 0.79 for F-Seq, MACS and Hotspot, respectively. Thus, the introduced recall measure to optimize peak calling parameters can explain 79% to 92% of the variation in the ${F}_{1}$-score. The results of this analysis are summarized in Fig. 3 where each dot represents one replicate of one cell line given a certain parameterization.

**Table 1:** Thresholds used for each peak caller in the analyses throughout the manuscript.

Peak Caller	Thresholds
F-Seq (version 1.84)	0.5, 1, 2, 3, 4, 5, 6, 7
MACS (version 2.1.0.20140616)	0.01, 0.05, 0.075, 0.1, 0.15, 0.2
Hotspot (version 5.1)	0.01, 0.05, 0.075, 0.1, 0.15, 0.2

For MACS and Hotspot, thresholds correspond to false discovery rate (FDR) and in case of F-Seq to z-score thresholds. Z-score thresholds are negatively correlated with FDR. Thus, small values correspond to high FDR thresholds and large values to low FDR thresholds.

While the coefficients of determination (adj. R-square values) provide quantitative measures of the agreement between the proposed recall measure and the ${F}_{1}$-scores, we are eventually interested in ranking the observations to identify the optimal parameter set. Following this rationale, we derived rankings of the parameters based on recall and ${F}_{1}$-score for each peak caller in each cell line of the gold standard. Comparison of the results yields in all cases a perfect agreement (Pearson’s r: 1.00, p-value: 0) meaning that the rankings obtained by our proposed recall measure are equal to the rankings obtained by ${F}_{1}$-scores. This eventually proves the ability of our method to identify the optimal parameter settings for each peak caller even in the absence of a ground truth.

## Discussion

The interpretation and detection of regions of enriched signals in accessible chromatin assays – e.g. FAIRE-seq or DNase-seq – suffer from different inherent limitations. On the one hand, computational data interpretation of DNase-seq experiments is easier in comparison to FAIRE-seq due to a higher signal-to-noise ratio^39^. However, on the other hand, a recent study performing a thorough assessment of most commonly used peak callers including MACS^24^, ZINBA^23^, F-Seq^22^ and Hotspot^16, 21^ revealed that computational methods to interpret DNase-seq data have also important limitations. In particular, the outcome of these methods heavily relies on the selection of many parameters, which in turn depends on the experimental conditions. More importantly, a systematic comparison revealed that all peak calling algorithms result in false discovery rates higher than 0.7 to obtain acceptable true positive rates^25^. Among all compared methodologies, Hotspot showed the least sensitivity to its parameter settings. The same study found that the overlap of detected peaks - i.e. accessible regions - of the four studied peak callers amounts to only 11%, which hampers the selection of the most appropriate tool for the analysis^25^. Hence, in order to overcome these limitations, we developed a hierarchical classification tree model correlating transcriptomics and the accessibility of gene-regulatory chromatin regions from a set of cell types and cell lines.

We compared our method and three of the most widely used peak calling algorithms, F-Seq^22^, MACS^24^ and Hotspot^16, 21^, against gold standard datasets obtained for six different cell lines from ENCODE^13^. It is to note, that we excluded ZINBA^23^ from our comparison due to its extended runtime^25^. In all six gold standard datasets, our methodology obtained strong ${F}_{1}$ - scores – computed as the harmonic mean of precision and recall – and outperformed all peak calling algorithms. Since our methodology is trained on gene expression data, we examined misclassified genes, especially with low expression values, to identify potential biases. In particular, our analysis focused on characteristics such as the GC content of genes^40, 41^, which is negatively correlated with the genes methylation level, and highlights the ability of our method to distinguish gene-regulatory regions located in heterochromatin from those regulated by other epigenetic or transcriptional mechanisms.

Peak calling algorithm, such as F-Seq, MACS and Hotspot, have not only been used to study whether genes are transcriptionally or epigenetically regulated but allow for further functional analyses including the identification of distal regulatory elements^42^. However, all peak callers exhibit a vast amount of parameters that are ultimately influencing the identified hypersensitive sites and an optimal setting is not a priori known. While experimental procedures, such as qPCR, have been previously used to validate putative DNase hypersensitive sites^43^, an optimal computational strategy, that eventually saves time and resources, still does not exist. The method presented in this work can identify optimal sets of parameters for peak calling algorithms by comparing the observations of these tools – based on certain parameter settings – with respect to the predictions generated by our method. Moreover, it was able to accurately rank all analyzed parameter settings of all peak callers in regard to the six gold standard datasets, which allows the selection of an optimal set of parameters without prior knowledge.

Another important aspect considers the experimental requirements for obtaining reliable DNase-seq experiments. Typically, genome-wide identification of hypersensitive sites requires millions of cells^21, 44^ not available for all samples, such as patient derived cancer cells. Thus, further exploring the possibilities to increase the precision of predicting accessible and inaccessible chromatin regions could help to overcome this experimental limitation.

We believe that the method presented in this work offers a valuable tool for identifying the chromatin landscape of gene-regulatory regions. It can further assist current peak calling methodologies in more reliably identifying gene regulatory regions not undergoing transcription throughout the genome by optimizing their parameter settings. The application of the hierarchical classification tree model is not limited to the prediction of accessible and inaccessible gene-regulatory chromatin regions from bulk gene expression data alone, but can be further extended. For example, chromatin interaction data from Hi-C experiments^45^ can be used as additional input data and further improves the performance for classifying lowly expressed genes (see Supplementary File S2 for analysis details and results). Further work will be needed to extend it to single cell expression and accessibility data and evaluate the performance of the method in these datasets.

## Methods

The present study infers the gene-level chromatin accessibility from transcriptomics data based on a developed hierarchical classification tree model. Given transcriptomics data of 18 human RNA-Seq and corresponding DNase-seq experiments, the model learns the relationship of expression levels and binary gene-level chromatin accessibility and can be readily applied to new, unseen transcriptomics datasets. The predictions are validated by gold standard datasets of genes located in accessible and inaccessible chromatin. While genes in accessible chromatin are defined by protein binding events in their proximity revealed by transcription factor binding site (TFBS) ChIP-seq, genes in inaccessible chromatin are characterized by heterochromatin associated genomic states of histone modifications. In the remainder of this section, we will outline the methodological details of the aforementioned study design.

### Training dataset preparation

We prepared a dataset including 18 human (Human Genome Version 19, hg19) RNA-seq and corresponding DNase-seq samples to train our methodology, all of which are annotated in ENCODE^13^. These samples include various cell line and cell type samples from different parts of the organism, ranging from alveolar basal epithelial cells over skeletal muscle cells up to embryonic stem cells. A detailed summary of all datasets can be found in Supplementary Table S1.

Reads alignments to the corresponding reference genome of DNase-seq samples were obtained from ENCODE^13^ (http://hgdownload.cse.ucsc.edu/goldenPath/hg19/encodeDCC/wgEncodeUwDnase/), and DNaseI sensitive zones (HotSpots) were identified using the HotSpot algorithm (version 5.1)^21, 46^ with default parameters. Specifically, a false discovery rate (FDR) threshold of 1.0% was imposed on all identified sites. We derived a binary assignment of the accessibility of a gene by calling it ‘open’ if the gene-coding region (including 3′ and 5′ UTR) or promoter, defined by annotations in HOMER^47^, contains at least one hotspot,and ‘closed’ otherwise. For RNA-Seq experiments, alignments of long PolyA+ RNA-seq samples were obtained from ENCODE^13^ (CSHL Long RNA-seq track, see Supplementary Table S1 for accession numbers). Transcript abundance was estimated using HOMER v4.8.2^47^ (homo sapiens accession version 5.8) by first creating a tag directory using the makeTagDirectory command with default parameters. Second, gene expression was quantified in FPKM format by running the analyzeRepeats program. Only reads mapping to exons on either strand were counted and different isoforms were condensed to the gene level.

### Performance analysis on the basis of a gold standard dataset

The performance of our method and three peak calling algorithms was assessed over a wide range of false discovery rate (FDR) cutoffs for MACS and Hotspot and z-score cutoffs for F-Seq (Table 1). Each method was evaluated on gold standard datasets of six cell types (A549, GM12878, H1-hESC, HeLa-S3, HepG2 and K562) comprised of reference sets for accessible and inaccessible genes. We compiled a set of regulatory regions from cell type specific transcription factor binding site (TFBS) ChIP-seq experiments deposited in ENCODE^13^. Each regulatory region was annotated using HOMER’s^47^ annotatePeaks command with default parameters and each gene overlapping these regions was subsequently added to the reference set of accessible genes. Genes containing intragenic enhancers bound by a transcription factor are therefore considered to be accessible which is motivated by the fact that intragenic enhancers act as alternative promoters^48^ and thus the same reasoning applies as for typical promoter regions (see previous section). Inaccessible genes, on the other hand, are defined by ChromHMM^29^, a computational tool widely used to integrate epigenetics experimental data to segment the genome into chromatin states. We used precompiled segmentations from the Roadmap Epigenomics project^11^ based on five histone modifications – i.e. H3K4me1, H3K4me3, H3K36me3, H3K9me3 and H3K27me3. Segments that are annotated to H3K9me3 alone or a combination of H3K36me3, H3K4me3 and H3K9me3 are then defined as heterochromatic regions. Consequently, genes overlapping with at least one of these segments are then added to the reference set of inaccessible genes.

Based on the gold standard dataset of accessible and inaccessible genes, we compared the performance of our method and peak calling algorithms by means of the harmonic mean of precision and recall defined as the ${F}_{1}$ – score:

$${F}_{1}=2\cdot \frac{precision\cdot recall}{precision+recall}$$

Assuming that accessible genes are the positive class and inaccessible genes the inaccessible genes, precision can be defined as the fraction of true positives among the predicted positives, $\frac{{TP}}{{TP}+{FP}}$, and recall as the ratio of true positives among all real positives, $\frac{{TP}}{{TP}+{FN}}$ ^25^. In the context of predicting accessible and inaccessible genes, these definitions can be described as follows. Precision represents the ratio of correctly predicted accessible genes overlapping the accessible genes in the gold standard dataset. Similarly, recall is the ratio of predicted accessible genes that overlap the gold standard dataset over all accessible genes in the gold standard dataset.

### Hierarchical classification model fitting

For each training dataset we fit a classification tree model consisting of three hierarchically combined layers, denoted as ${L}_{B},{L}_{M}\,\text{and}\,{L}_{U}$ for the bottom, middle and upper layer trees, respectively. The ${L}_{B}$ classifiers operate directly on the gene expression/open chromatin pair values. In particular, gene expression values correspond here to FPKM values obtained from RNA-Seq data. Open chromatin of the corresponding gene is then assigned a binary value depending on whether the gene-coding region or promoter contains a called peak.

In a next step, we fitted one thousand ${L}_{B}$ classification trees for each sample. Each tree is built on a uniform sample of 1000 expression values/observations without replacement. The number of ${L}_{B}$ trees is determined by the empirical number of random samples needed to select each gene at least once and is multiplied by four to obtain well mixed training datasets. Especially for low expression, similar expression values might have different corresponding accessibility values resulting in too fine-grained conditions in the classification tree – e.g. conditions on the fourth decimal place of an expression value. Drawing samples of 1000 expression value/observation pairs is addressing this issue by reducing the number of similar expression values while preserving the distribution of the initial sample. In a next step, the predictions of all ${L}_{B}$ trees of a sample in the training dataset are combined by another classification tree (${L}_{M}$ classifier), relating the prediction scores with open chromatin information, which is equivalent to learning the separations of scores. We thus obtain as many ${L}_{M}$ classifiers as there are samples in the respective training dataset. Based on the identified DNase peaks, all training samples show an uneven ratio of genes encoded in hetero- and euchromatin, biasing the classification tree fitting. To account for the underlying imbalance, we employed a RUSBoost algorithm^49^ as the basis of each ${L}_{M}$ classifier. Due to the impossibility of processing all combinations of random subsets, a principal component analysis (PCA) was conducted to account for oversampling of the same values resulting in biased predictions of our methodology. Specifically, those principal components are used as predictors of the ${L}_{M}$ explaining at least 95% of the variance. Finally, the predictions of all ${L}_{M}$ classifiers – i.e. the predictions of all samples – are combined within a single RUS-Boost classifier (${L}_{U}$ classifier) to obtain the final predictions.

In contrast to ${L}_{M}\,\text{and}\,{L}_{U}$ trees, ${L}_{B}$ trees are grown iteratively starting from a single node containing all observations following the *fitctree* implementation in MATLAB. Notably, besides using gene expression as a predictor we employed the Mahalanobis distance^50^ of the expression value to the distribution of accessible and inaccessible genes, respectively. For expression value ${e}_{i}$ the Mahalanobis distance to the empirical distribution $X$ is defined as $d({e}_{i})=({e}_{i}-{\mu }_{X})\bullet {S}_{X}^{-1}\bullet ({e}_{i}-{\mu }_{X})^{\prime}$ where ${S}_{X}^{-1}$ is the inverse of the sample covariance matrix of X.

### Data Availability

The source code of the method as well as example input data can be accessed online (https://github.com/saschajung/ChromAccPrediction).

## Electronic supplementary material

Supplementary Information

## References

[1] Li X-Y (2011). The role of chromatin accessibility in directing the widespread, overlapping patterns of Drosophila transcription factor binding. Genome Biol..

[2] Lickwar CR, Mueller F, Hanlon SE, McNally JG, Lieb JD (2012). Genome-wide protein-DNA binding dynamics suggest a molecular clutch for transcription factor function. Nature.

[3] Kasowski M (2013). Extensive variation in chromatin states across humans. Science (80-.).

[4] Kilpinen H (2013). Coordinated effects of sequence variation on DNA binding, chromatin structure, and transcription. Science (80-.).

[5] McVicker G (2013). Identification of genetic variants that affect histone modifications in human cells. Science (80-.).

[6] Lavin Y (2014). Tissue-resident macrophage enhancer landscapes are shaped by the local microenvironment. Cell.

[7] Apostolou E, Hochedlinger K (2013). Chromatin dynamics during cellular reprogramming. Nature.

[8] Gaspar-Maia A, Alajem A, Meshorer E, Ramalho-Santos M (2011). Open chromatin in pluripotency and reprogramming. Nat Rev Mol Cell Biol.

[9] Lara-Astiaso D (2014). Immunogenetics. Chromatin state dynamics during blood formation. Science (80-.).

[10] Gjoneska E (2015). Conserved epigenomic signals in mice and humans reveal immune basis of Alzheimer’s disease. Nature.

[11] Roadmap Epigenomics C (2015). Integrative analysis of 111 reference human epigenomes. Nature.

[12] Bernstein BE (2010). The NIH Roadmap Epigenomics Mapping Consortium. Nat Biotechnol.

[13] Dunham I (2012). An integrated encyclopedia of DNA elements in the human genome. Nature.

[14] Boyle AP (2008). High-resolution mapping and characterization of open chromatin across the genome. Cell.

[15] Song L (2011). Open chromatin defined by DNaseI and FAIRE identifies regulatory elements that shape cell-type identity. Genome Res..

[16] Thurman RE (2012). The accessible chromatin landscape of the human genome. Nature.

[17] Mercer TR (2013). DNase I-hypersensitive exons colocalize with promoters and distal regulatory elements. Nat Genet.

[18] Neph S (2012). Circuitry and dynamics of human transcription factor regulatory networks. Cell.

[19] Buenrostro JD, Giresi PG, Zaba LC, Chang HY, Greenleaf WJ (2013). Transposition of native chromatin for fast and sensitive epigenomic profiling of open chromatin, DNA-binding proteins and nucleosome position. Nat Methods.

[20] Hesselberth JR (2009). Global mapping of protein-DNA interactions *in vivo* by digital genomic footprinting. Nat Methods.

[21] John S (2011). Chromatin accessibility pre-determines glucocorticoid receptor binding patterns. Nat. Genet..

[22] Boyle AP, Guinney J, Crawford GE, Furey TS (2008). F-Seq: a feature density estimator for high-throughput sequence tags. Bioinformatics.

[23] Rashid NU, Giresi PG, Ibrahim JG, Sun W, Lieb JD (2011). ZINBA integrates local covariates with DNA-seq data to identify broad and narrow regions of enrichment, even within amplified genomic regions. Genome Biol..

[24] Zhang Y (2008). Model-based analysis of ChIP-Seq (MACS). Genome Biol..

[25] Koohy H, Down TA, Spivakov M, Hubbard T (2014). A Comparison of Peak Callers Used for DNase-Seq Data. PLoS One.

[26] He, Y. *et al*. Genome-wide mapping of DNase I hypersensitive sites and association analysis with gene expression in MSB1 cells. *Front. Genet*. **5**, (2014). 10.3389/fgene.2014.00308 PMC4195362 25352859

[27] Moncunill, V. *et al*. Comprehensive characterization of complex structural variations in cancer by directly comparing genome sequence reads. *Nat. Biotechnol*. **32**, 1106–1112 (2014). 10.1038/nbt.3027 25344728

[28] Malhotra A, Shibata Y, Hall IM, Dutta A (2013). Chromosomal structural variations during progression of a prostate epithelial cell line to a malignant metastatic state inactivate the NF2, NIPSNAP1, UGT2B17, and LPIN2 genes. Cancer Biol. Ther..

[29] Ernst J, Kellis M (2012). ChromHMM: automating chromatin-state discovery and characterization. Nat Methods.

[30] Chantalat S (2011). Histone H3 trimethylation at lysine 36 is associated with constitutive and facultative heterochromatin. Genome Res..

[31] Vogel MJ (2006). Human heterochromatin proteins form large domains containing KRAB-ZNF genes. Genome Res..

[32] Blahnik KR (2011). Characterization of the Contradictory Chromatin Signatures at the 3′ Exons of Zinc Finger Genes. PLoS One.

[33] Haltaufderhyde KD, Oancea E (2014). Genome-wide transcriptome analysis of human epidermal melanocytes. Genomics.

[34] Rau A, Gallopin M, Celeux G, Jaffrezic F (2013). Data-based filtering for replicated high-throughput transcriptome sequencing experiments. Bioinformatics.

[35] Trakhtenberg EF (2016). Cell types differ in global coordination of splicing and proportion of highly expressed genes. Sci. Rep..

[36] Meissner A (2008). Genome-scale DNA methylation maps of pluripotent and differentiated cells. Nature.

[37] Djebali S (2012). Landscape of transcription in human cells. Nature.

[38] Iyer MK (2015). The landscape of long noncoding RNAs in the human transcriptome. Nat. Genet..

[39] Tsompana M, Buck MJ (2014). Chromatin accessibility: a window into the genome. Epigenetics Chromatin.

[40] Zhang Y (2009). DNA Methylation Analysis of Chromosome 21 Gene Promoters at Single Base Pair and Single Allele Resolution. PLoS Genet..

[41] Newell-Price J, Clark AJL, King P (2000). DNA Methylation and Silencing of Gene Expression. Trends Endocrinol. Metab..

[42] Crawford GE (2005). Genome-wide mapping of DNase hypersensitive sites using massively parallel signature sequencing (MPSS). Genome Res..

[43] Crawford GE (2006). DNase-chip: a high-resolution method to identify DNase I hypersensitive sites using tiled microarrays. Nat. Methods.

[44] Song L, Crawford GE (2010). DNase-seq: A High-Resolution Technique for Mapping Active Gene Regulatory Elements across the Genome from Mammalian Cells. Cold Spring Harb. Protoc..

[45] Lieberman-Aiden E (2009). Comprehensive mapping of long-range interactions reveals folding principles of the human genome. Science.

[46] Sabo PJ (2004). Discovery of functional noncoding elements by digital analysis of chromatin structure. Proc. Natl. Acad. Sci..

[47] Heinz S (2010). Simple combinations of lineage-determining transcription factors prime cis-regulatory elements required for macrophage and B cell identities. Mol. Cell.

[48] Kowalczyk MS (2012). Intragenic Enhancers Act as Alternative Promoters. Mol. Cell.

[49] Seiffert C, Khoshgoftaar TM, Van Hulse J, Napolitano A (2010). RUSBoost: A hybrid approach to alleviating class imbalance. IEEE Trans. Syst. Man, Cybern. Part ASystems Humans.

[50] Mahalanobis PC (1936). On the generalized distance in statistics. Proc. Natl. Inst. Sci. India.

