# Supplementary material for: Prediction of Chromatin Accessibility in Gene-Regulatory Regions from Transcriptomics Data
Source: Sci Rep. 2017 Jul 5;7:4660. doi: 10.1038/s41598-017-04929-6 (PMC5498635; doi:10.1038/s41598-017-04929-6)
Supplement: Supplementary file 1 — Supplementary Information [file 41598_2017_4929_MOESM1_ESM.pdf]

Prediction of Chromatin Accessibility in Gene-Regulatory Regions from Transcriptomics Data

Sascha Jung[1], Vladimir Espinosa Angarica[1], Miguel A. Andrade-Navarro[2,3], Noel Buckley[4], Antonio del Sol[1,*]

Supplementary File S1: Reproducibility and Validation of the Hierarchical Classification Model

The validation of a predictive model is crucial to assess its sensitivity to the training data and its ability to predict unseen data. In order to quantify the aforementioned attributes, we performed Leave-one-out cross-validation that partitions the data into disjoint subsets, trains the model with all but one of these sets and assesses the model performance based on the hold out set.

In a first assessment, the model was trained with 17 out of 18 cell line samples and predictions where carried out on the hold out sample. The binary predictions as well as the scores, i.e. probabilities, for being accessible and inaccessible are then compared to the model trained with all samples by means of Pearson correlation (Fig. S1.1A). We observe that the median correlation between the full model's and leave-one-out predictions is 0.984 for the binary classifications (red) and 0.997 for the scores (green) while the individual correlations are always greater than 0.95. Next, we sought whether these results can be confirmed when the training set is partitioned by chromosome, i.e. all but one chromosome is used for training the model while predictions are carried out on the hold out chromosome. Here, we again distinguished the actual binary classification into accessible and inaccessible chromatin regions and the scores for being in the respective classes. The correlations of the binary predictions (Fig. S1.1B) and the scores (Fig. S1.1C) obtained with the full model and the one trained with all but one chromosome validate the low sensitivity of our model to the training samples. The median binary correlations per chromosome range from 0.972 to 1 while the individual correlations are all greater than 0.947. Similarly, the median correlations of scores range from 0.987 to 0.999 with individual correlations greater than 0.98. Altogether, these results are in agreement with the previous assessment of leave-

one-out cross-validation based on samples and underline our model's insensibility towards the training set and supports our hypothesis that the model is not overfit.

Figure S1.1 Correlation of cross-validation samples with full model predictions

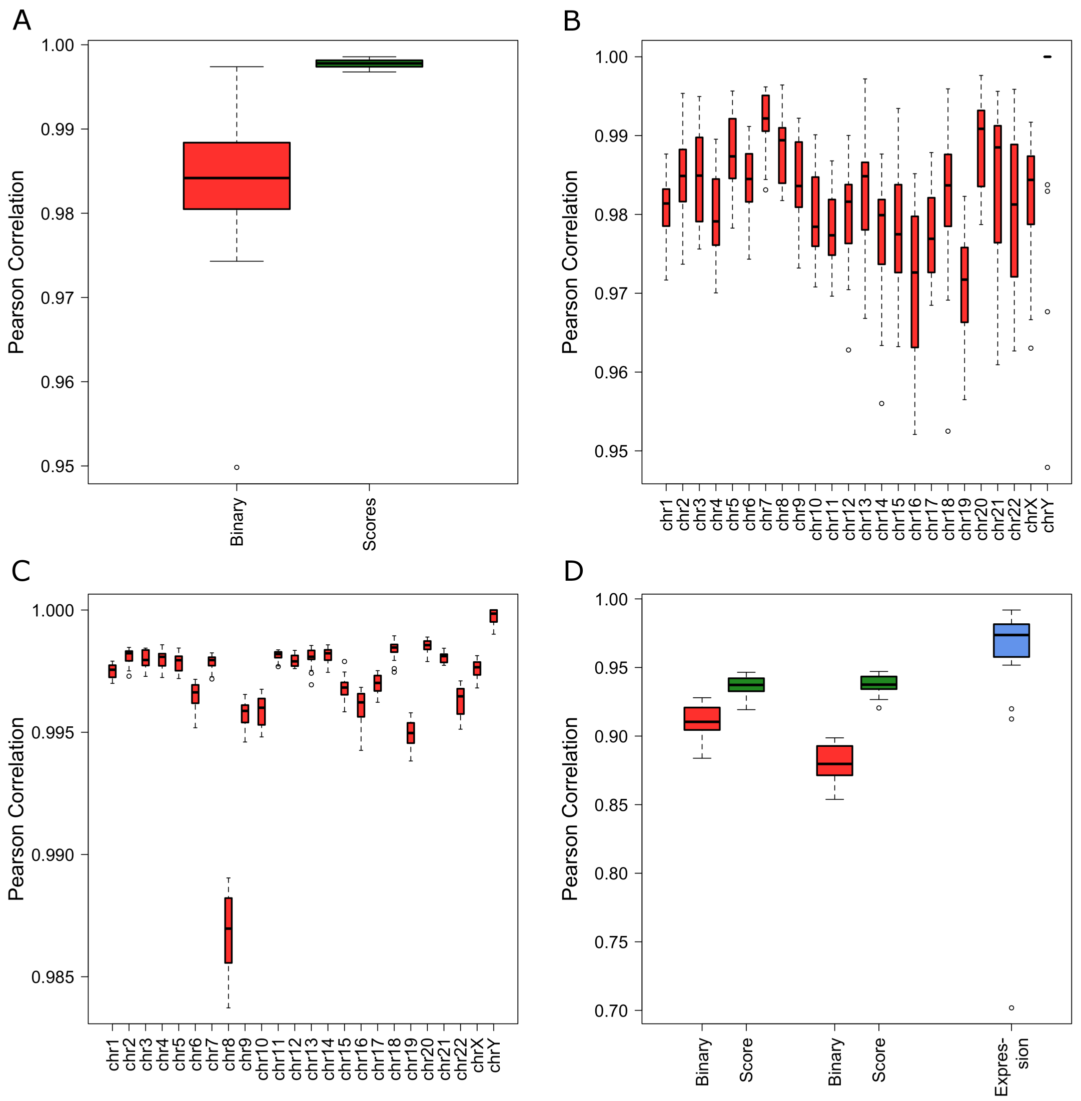

The correlation of different cross-validation assays with the predictions of the full model trained with all 18 samples. (A) Pearson correlation of the binary Leave-one-sample-out predictions with the full model and the corresponding correlations of the scores. (B) Pearson correlation of the binary Leave-one-chromosome-out predictions with the full model. (C) Pearson correlation of the scores associated to the binary Leave-one-chromosome-out predictions with the full model. (D) Reproducibility analysis results of the predictions with different replicates. The model was trained with the first replicates (left boxes) and second replicates (middle boxes) and the correlations of the predictions for both replicates were assessed in both models. Correlations based on binary predictions are shown in red and correlations based on scores are shown in green. The blue box shows the correlation between replicates of the same cell type of the gene expression data.

At last, we investigated the reproducibility of the predictions in different replicates of the same cell type/line. We thus collected a second replicate for each cell line included in the original training dataset generated in the same lab and computed the correlation of replicates of the same cell line (Fig. S1.1D). As expected, the median correlation between replicates is 0.97 with HeLa-S3 cells being an outlier with correlation 0.7 (blue box). Of note, the obtained dataset for HMEC cells was of poor quality showing mostly 0 FPKM for the gene and was therefore excluded from the analysis. We then trained the model with the first replicates, predicted the second replicates and compared the agreement with the predictions of the first replicates (left boxes), and vice versa (middle boxes). Here, red boxes again represent the correlations between binary accessibility predictions and green boxes the correlations between the prediction scores. Our results show median correlations of 0.91 and 0.88, respectively, in the binary case indicating high reproducibility of the results. Interestingly, the correlations of the scores do not show any difference (medians: 0.937), which indicates that the threshold for binarizing the scores in both cases have to be different. However, the optimal threshold cannot be determined for unseen data and is thus kept at 0.5, which still results in very high correlations above 0.85 after discretization.

Supplementary File S2: Prediction of Chromatin Accessibility from Transcriptomics and Hi-C Data

The hierarchical classification tree model presented in the main text predicts accessible and inaccessible gene-coding regions solely based on transcriptomics data. However, the model is generally able to incorporate an arbitrary number of predictors for enhancing its predictive power. We thus asked whether we are able to improve the predictive power of our method by incorporating chromatin interaction data from Hi-C experiments[1]. These experiments are able to capture 3D chromatin interactions that are possibly far away in 1D genomic distances and can be readily used to divide the genome into two compartments harboring active and inactive regions, respectively[1]. In particular, the compartmentalization is achieved by dividing the genome into windows of a certain size, creating an interaction matrix of the signal data and performing principal component analysis (PCA) on the interaction matrix. The sign of the first principal component can then be used to classify the different compartments, i.e. all regions having the same sign belong to the same compartment.

For predicting the chromatin accessibility, as detailed in the main manuscript, we collected Hi-C datasets of 10 different cell lines, which are also included in the 18 training datasets used in the main text, segmented the genome into bins of 50kb and computed the first principal component on the resulting interaction matrix (see Methods section below for details). Genes are then associated to the value of the first principal component of the region they are overlapping the most with. In addition to the raw gene expression values and the Mahalanobis distances to accessible and inaccessible genes, the values of the first principal component are included as a predictor for the hierarchical classification tree model.

After training the model with this dataset, we assessed the F_1- score of the predictions with respect to the gold standard dataset described in the main text and compared them to the scores obtained with the full model of 18 datasets not including Hi-C experiments (see Fig. S2.1). The results show average improvements of 0.03 in the classification of lowly expressed genes, i.e. when considering expression cutoffs of 0.01 and below, but at the same time on average 0.02 lower scores for genes that are clearly expressed, i.e. expression cutoff above 2 FPKM. Here, the black and red lines show the F_1- scores for the predictions with and without Hi-C data, respectively. However, the interpretation of the results has to be taken with care since the size of the training dataset is much smaller when using Hi-C data and thus might be a possible explanation of the small differences. Nevertheless, the results already suggest that the incorporation of chromatin interaction data helps the model to more accurately classify accessible and inaccessible gene-coding chromatin regions.

Figure S2.2. Comparison of the model trained with and without Hi-C data

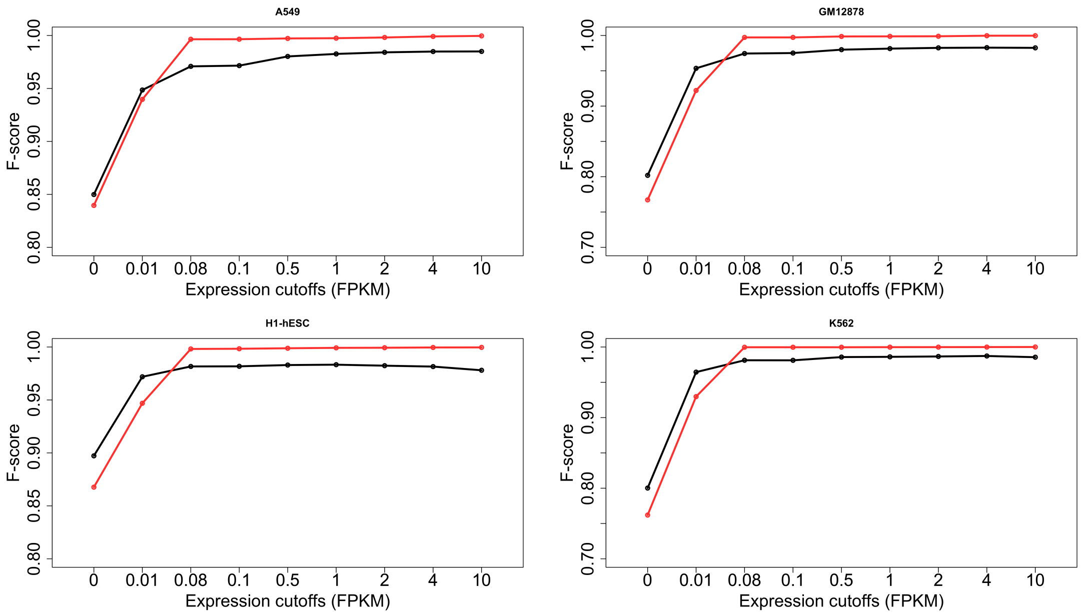

Comparison of F_1-scores after applying lower expression cutoffs in four gold standard datasets of our predictions with (black line) and without (red line) using Hi-C data. Only genes that are more expressed than the cutoff were taken into account for the calculation of F_1-scores (0 cutoff representing all genes). While using Hi-C data shows on average 0.03 higher scores for the whole dataset, the performance is slightly decreased (on average 0.02 lower scores) when considering only clearly expressed genes.

Methods

Hi-C data acquisition and analysis

We collected 10 publicly available Hi-C datasets for cell types and cell lines already included in the main training dataset. An overview of the datasets can be found in Table S2.1. Raw reads were downloaded in fastq format and aligned to the hg19 reference genome using Bowtie 2[2] with the –very-sensitive option. Aligned reads were subsequently filtered for alignments with MAPQ value greater than 30 and uniquely mapping reads. Further processing was performed using HOMER[3]. Tag directories were created for each dataset and filtered for uninformative reads. Specifically, we removed reads that (i) are likely continuous genomic fragments or re-ligation events (-removePEbg), (ii) have a 5-fold higher tag density than the average in a 10kb window (-remove spikes 10000 5), (iii) are not in the vicinity of the restriction site used for the assay (-restrictionSite AAGCTT) and (iv) form a self ligation with adjacent restriction sites. Finally, principal component analysis was performed using the tag directories as input with a resolution of 50kb (-res 50000) and a window size of 100kb for the background model (-superRes 100000).

Table S2.1. Hi-C datasets used for the analysis

Cell line	Availability
A549	Encode project (ENCSR662QKG)
GM12878	SRA (SRR1658570)
H1-hESC	SRA (SRR1030718, SRR1030719, SRR1030720, SRR1030721)
HMEC	SRA (SRR1658680)

HUVEC	SRA (SRR1658712)
IMR90	SRA (SRR1658673)
K562	SRA (SRR1658693)
MCF7	SRA (SRR1909070)
NHEK	SRA (SRR1658689)
SK-N-SH (RA)	SRA (SRR2106508, SRR2106509, SRR2106510)

Hi-C datasets used in the training dataset with corresponding accession numbers in either the ENCODE project (A549 only) or the sequence read archive (SRA). SRA accessions are given per run and can be searched for at https://www.ncbi.nlm.nih.gov

Supplementary Figure S1: Validation of de novo predicted accessible genes

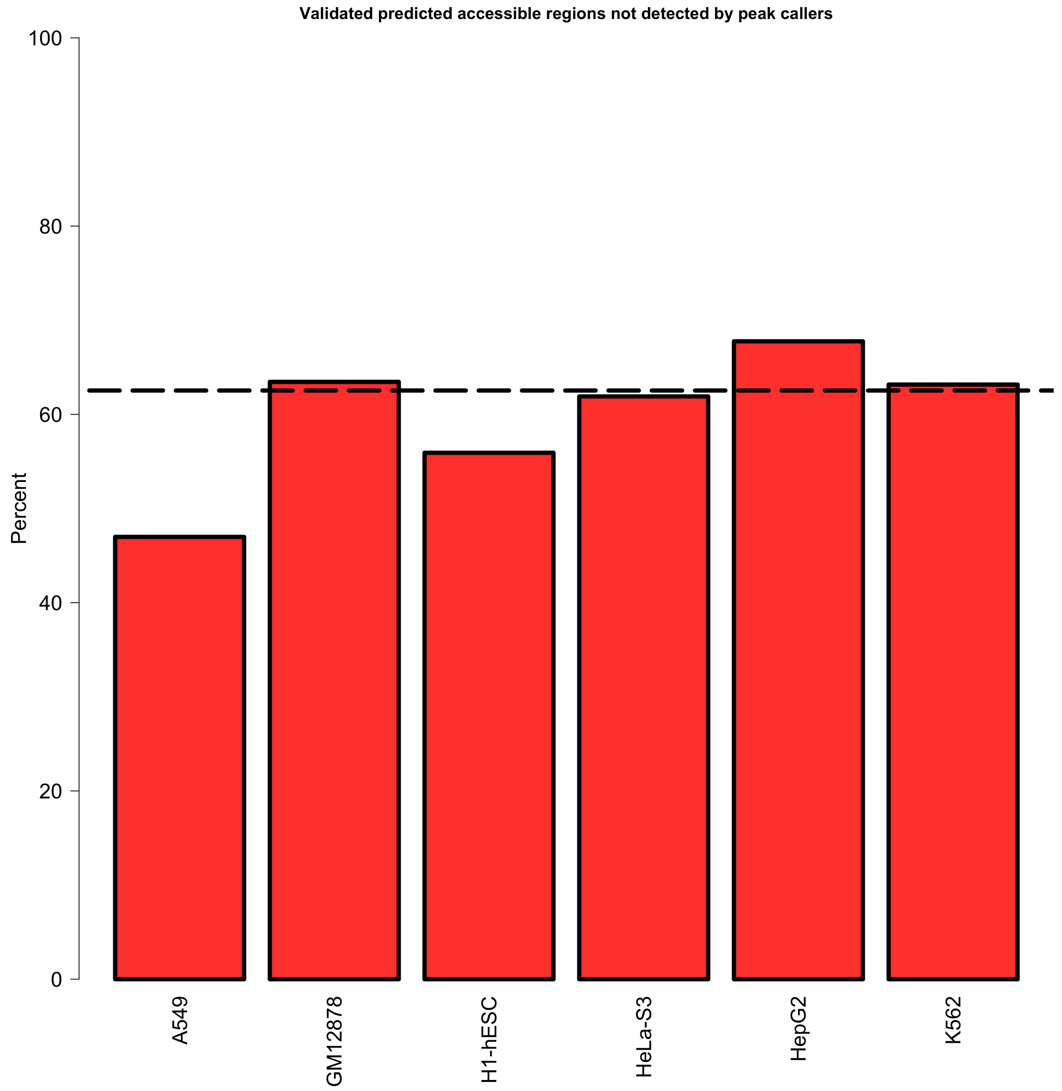

Percentage of validated accessible genes that are predicted by the model and not detected by peak calling methods. Validation was performed on the basis of the TFBS ChIP-seq experiments in the gold standard datasets from ENCODE. Bars represent the percentage of genes showing a binding event, and as such are deemed to be accessible, of all predicted accessible genes that are not detected by peak callers. Between 49% (A549) and 69% of de novo predictions could be validated (median 62%, dashed line).
